# Supplementary material for: Nonlinear genomic selection index accelerates multi-trait crop improvement
Source: Nat Commun. 2026 Feb 20;17:1991. doi: 10.1038/s41467-026-69890-3 (PMC12932720; doi:10.1038/s41467-026-69890-3)
Supplement: Supplementary file 1 — Supplementary Information [file 41467_2026_69890_MOESM1_ESM.pdf]

Nonlinear genomic selection index accelerates multi-trait crop improvement

Cerón-Rojas *et al.*

Supplementary Method 1. Quadratic phenotypic selection index theory

The quadratic net genetic merit can be writing as

$$H_q = t(\mathbf{w})\mathbf{g} + t(\mathbf{g})\mathbf{W}\mathbf{g}, \qquad (\mathbf{1})$$

$$= \sum_{i=1}^{t} w_i g_i + \sum_{i=1}^{t} \alpha_{ii} g_i^2 + \sum_{i=1}^{t}\sum_{j=1+i}^{t} \alpha_{ij} g_i g_j$$

where $t(\mathbf{w}) = [w_1 \quad w_2 \quad \cdots \quad w_t]$ and $t(\mathbf{g}) = [G_1 \quad G_2 \quad \ldots \quad G_t]$ denote the transpose of the vector of known economic weights **w** and the vector of breeding values **g** which has multivariate normal distribution, null mean, and covariance matrix **G**. In addition, matrix **W** can be written as

$$\mathbf{W} = \begin{bmatrix} \alpha_{11} & 0.5\alpha_{12} & \cdots & 0.5\alpha_{1t} \\ 0.5\alpha_{12} & \alpha_{22} & \cdots & 0.5\alpha_{2t} \\ \vdots & \vdots & \ddots & \vdots \\ 0.5\alpha_{1t} & 0.5\alpha_{2t} & \ldots & \alpha_{tt} \end{bmatrix},$$

where the ith diagonal values α_{ii} (i =1,2, …, t) is the relative economic weight associated to the genetic value of the ith squared trait and $0.5\alpha_{ij}$ (i, j =1,2, …, t) is the economic weight of the cross products between the genetic values of traits i and j. Note that the elements of matrix **W** might be different to the elements of vector **w**[1]. The expectation and variance of Supplementary Equation **1** are $E(H_q) = tr(\mathbf{WG})$ and $Var(H_q) = t(\mathbf{w})\mathbf{G}\mathbf{w} + 2tr[\mathbf{WGWG}]$, respectively, where $tr(\cdot)$ denotes the trace function of matrices inside parenthesis.

The quadratic phenotypic selection index (QPSI)

This index is a predictor of Supplementary Equation **1** and can be written as

$$I_q = t(\mathbf{b})\mathbf{y} + t(\mathbf{y})\mathbf{B}\mathbf{y} \qquad (\mathbf{2})$$

$$= \sum_{i=1}^{t} b_i y_i + \sum_{i=1}^{t} \beta_{ii} y_i^2 + \sum_{i=1}^{t}\sum_{j=1+i}^{t} \beta_{ij} y_i y_j$$

where $t(\mathbf{b}) = [b_1 \quad b_2 \quad \cdots \quad b_t]$ and $t(\mathbf{y}) = [Y_1 \quad Y_2 \quad \cdots \quad Y_t]$ denote the transpose of the vector of linear coefficients **b** and the vector transpose of the adjusted and centered phenotypic values, **y**, respectively, which has multivariate normal distribution, zero mean ($\boldsymbol{\mu} = \mathbf{0}$), and covariance matrix **P**, whereas

$$\mathbf{B} = \begin{bmatrix} \beta_{11} & 0.5\beta_{12} & \cdots & 0.5\beta_{1t} \\ 0.5\beta_{12} & \beta_{22} & \cdots & 0.5\beta_{2t} \\ \vdots & \vdots & \ddots & \vdots \\ 0.5\beta_{1t} & 0.5\beta_{2t} & \dots & \beta_{tt} \end{bmatrix}$$

is a matrix where the ith diagonal values β_{ii} ($i = 1,2, \ldots, t$) is the index weight for the square of the ith phenotypic value, and $0.5\beta_{ij}$ ($i, j = 1,2, \ldots, t$) is the index weight for the cross products between the ith and j^{th} trait phenotype values. Note that the elements of vector **b** are different to the elements of matrix **B** . In Supplementary Equation **2**, **b** is the vector of directional selection gradients and **B** is the matrix of nonlinear selection gradients. The diagonal elements (β_{ii}) of **B** are the stabilizing/disruptive selection gradient for trait i^{th}, and β_{ij} is a covariance or correlational selection gradient for traits i^{th} and j^{th}. In this case **b** measures the strength and direction of selection on each trait, whereas the stabilizing selection gradient indicates a preference for intermediate traits, and a disruptive selection gradient indicates a preference for both extreme traits over the intermediate ones. A negative **B** shows stabilizing selection, while a positive one shows disruptive selection. In the univariate and bivariate case, Supplementary Equation **2** reduces to

$I_i = b_i y_i + \beta_{ii} y_i^2$ and $I_{ij} = b_i y_i + b_j y_j + \beta_{ii} y_i^2 + \beta_{jj} y_j^2 + \beta_{ij} y_i y_j$,

respectively.

Minimizing the mean square prediction error (MSPE) of I_q

To obtain the vector ($\mathbf{b}$) and the matrix ($\mathbf{B}$) values that maximize the selection response and the correlation between I_q and H_q, we can minimize the mean square prediction error (MSPE), which is the expectation of the square difference between I_q and H_q[1] and can be written as

$$\Pi = E\langle\{[I_q - E(I_q)] - [H_q - E(H_q)]\}^2\rangle$$
$$= t(\mathbf{w})\mathbf{Gw} + 2tr(\mathbf{WGWG}) + 2tr(\mathbf{BPBP}) + t(\mathbf{b})\mathbf{Pb} - 2t(\mathbf{b})\mathbf{Gw} - 4tr(\mathbf{BGWG}), \quad (\mathbf{3})$$

where $t(\mathbf{w})$ and $t(\mathbf{b})$ have been defined earlier. To find the **b** and **B** values that minimize Supplementary Equation **3**, it is necessary to derivative this equation with respect to **b** and **B**, equate the result of the derivative to zero, and isolate **b** and **B**. The first partial derivatives result of Supplementary Equation **3** with respect to **b** and **B** when the vector of trait phenotypic mean values is null ($\boldsymbol{\mu} = \mathbf{0}$) are[1], respectively,

$$\mathbf{b} = \mathbf{P}^{-1}\mathbf{Gw} \text{ and } \mathbf{B} = \mathbf{P}^{-1}\mathbf{GWGP}^{-1}, \qquad (\mathbf{4})$$

which minimize Π. It is possible to show that the second partial derivatives of Supplementary Equation **3** respect to **b** and **B** are positive and then, in effect, Supplementary Equation **4** minimize Π.

The minimized Π value is

$$t(\mathbf{w})\mathbf{Gw} - t(\mathbf{b})\mathbf{Pb} + 2tr(\mathbf{WGWG}) - 2tr(\mathbf{BGWG}), \quad (\mathbf{5})$$

where $t(\mathbf{w})$, $t(\mathbf{b})$, and **W** have been defined earlier; $t(\mathbf{w})\mathbf{Gw} - t(\mathbf{b})\mathbf{Pb}$ is the LPSI MSPE (also called prediction error variance), whereas $2tr(\mathbf{WGWG}) - 2tr(\mathbf{BGWG})$ is the additional quadratic MSPE. Therefore, when $\mathbf{B} \neq \mathbf{W}$ and $[2tr(\mathbf{WGWG}) - 2tr(\mathbf{BGWG})] > 0$, the Supplementary Equation **5** (QPSI MSPE) should be higher than the LPSI MSPE. The accuracy of the estimated $2tr(\mathbf{WGWG}) - 2tr(\mathbf{BGWG})$values will depend of the estimated $\mathbf{B} = \mathbf{P}^{-1}\mathbf{GWGP}^{-1}$, $\mathbf{P}^{-1}$, and **G** values. In the asymptotic context $\mathbf{B} = \mathbf{P}^{-1}\mathbf{GWGP}^{-1}$ should tend to the **W** values because the

increase of the number of genotypes and environment where the traits are evaluated only affects matrices $\mathbf{P}^{-1}$ and $\mathbf{G}$, and then, the multi-trait heritability ($\mathbf{P}^{-1}\mathbf{G}$), which should tend to an identity matrix $\mathbf{I}_{t\times t}$. In this last case, the minimized Π value is zero, that is, $\Pi = 0$.

The square correlation between $\mathrm{I_q}$ and $\mathrm{H_q}$

Because $\mathrm{I_q}$ and $\mathrm{H_q}$ are quadratic, a good option to measure the relationship between $\mathrm{I_q}$ and $\mathrm{H_q}$ when $\mathbf{b} = \mathbf{P}^{-1}\mathbf{Gw}$ and $\mathbf{B} = \mathbf{P}^{-1}\mathbf{GWGP}^{-1}$, is the square correlation[2] between $\mathrm{I_q}$ and $\mathrm{H_q}$, that is,

$$\rho^2_{\mathrm{H_q I_q}} = \frac{\mathrm{t}(\mathbf{b})\mathbf{Pb}+2\mathrm{tr}[\mathbf{BGWG}]}{\mathrm{t}(\mathbf{w})\mathbf{Gw}+2\mathrm{tr}[\mathbf{WGWG}]}, \qquad (6)$$

where $\mathrm{t}(\mathbf{w})$, $\mathrm{t}(\mathbf{b})$, and $\mathbf{W}$ have been defined earlier; $\mathrm{Var}(\mathrm{I_q}) = \mathrm{t}(\mathbf{b})\mathbf{Pb} + 2\mathrm{tr}[\mathbf{BPBP}]$ is the variance of $\mathrm{I_q}$, and $\mathrm{Var}(\mathrm{H_q}) = \mathrm{t}(\mathbf{w})\mathbf{Gw} + 2\mathrm{tr}[\mathbf{WGWG}]$ is the variance of $\mathrm{H_q}$, whereas $\mathrm{Cov}(\mathrm{H_q}, \mathrm{I_q}) = \mathrm{t}(\mathbf{w})\mathbf{Gb} + 2\mathrm{tr}(\mathbf{BGWG})$ is the covariance between $\mathrm{I_q}$ and $\mathrm{H_q}$. Some authors[8] have showed that for $\mathbf{b} = \mathbf{P}^{-1}\mathbf{Gw}$ and $\mathbf{B} = \mathbf{P}^{-1}\mathbf{GWGP}^{-1}$, $\mathrm{Var}(\mathrm{I_q}) = \mathrm{Cov}(\mathrm{H_q}, \mathrm{I_q})$.

The QPSI selection response

The QPSI selection response[1] is

$$\mathrm{R_q} = \mathrm{k}\sqrt{\mathrm{t}(\mathbf{b})\mathbf{Pb} + 2\mathrm{tr}[\mathbf{BGWG}]}, \qquad (7)$$

where k is the selection intensity of the QPSI, whereas $\mathbf{b} = \mathbf{P}^{-1}\mathbf{Gw}$, and $\mathbf{B} = \mathbf{P}^{-1}\mathbf{GWGP}^{-1}$. Supplementary Equation **7** was originally obtained in the bivariate normal context[3], however, since k is usually a fixed constant, the relative genetic progress depends on $\mathrm{t}(\mathbf{b})\mathbf{Pb} + 2\mathrm{tr}[\mathbf{BPWG}]$ and not on k, so lack of normality is not so important for predicting relative genetic progress as for predicting absolute genetic progress[1].

In the asymptotic context, when $\mathbf{P}^{-1}\mathbf{G} = \mathbf{I}_{t\times t}$ (an identity matrix of order $t \times t$), Supplementary Equation **7** is equal to $\mathrm{R_q} = \mathrm{k}\sqrt{\mathrm{t}(\mathbf{w})\mathbf{Gw} + 2\mathrm{tr}[\mathbf{WGWG}]}$, the square root of the

variance of Supplementary Equation **1**, whereas Supplementary Equation **6** is equal to 1.0, as we would expect. Supplementary Equation **6** and **7** indicate that the LPSI and QPSI are related, and the only difference between them is the trace of the quadratic terms.

The QPSI expected genetic gain per trait

Since the covariance between I_q and $\mathbf{g}$ (Supplementary Equation **1**) is $\mathbf{Gb}^2$, the QPSI expected genetic gain per trait is the LPSI expected genetic gain per trait, that is,

$$\mathbf{E}_q = k\frac{\mathbf{Gb}}{\sigma_I}, \qquad (\mathbf{8})$$

where $\sigma_I = \sqrt{t(\mathbf{b})\mathbf{Pb}}$ is the standard deviation of the variance of the LPSI[4]. This means that as the **b** values are equal for linear and quadratic indices, the genetic response due to the quadratic term of the index is neglected in Supplementary Equation **8** because it uses a linear regression of the **g** on I_q^2.

Supplementary Method 2. Quadratic genomics selection index (QGSI) theory

This index is an application of the QLPSI theory (Supplementary Equation **1-8**) and the linear genomic selection index (LGSI)[5] to the genomics selection context. We shall construct the QGSI based on the vector of individual genomic breeding values, $\boldsymbol{\gamma}$, and its transpose, $t(\boldsymbol{\gamma}) = [t(\boldsymbol{\gamma}_1) \quad t(\boldsymbol{\gamma}_2) \quad \cdots \quad t(\boldsymbol{\gamma}_t)]$. In this context, vector $\boldsymbol{\gamma}_i$ (i =1, 2,…,t; t =number of traits) can be written as

$$\boldsymbol{\gamma}_i = \mathbf{X}\mathbf{u}_i \qquad \textbf{(9.1)}$$

where $\mathbf{X}$ is an $n \times m$ matrix (n=number of observations and m=number of markers in the population) of coded marker values ($2-2p$, $1-2p$ and $-2p$ for genotypes AA, Aa, and aa, respectively; p is the frequency of allele A, and $1-p$ is the frequency of allele a) associated with the additive effects of the quantitative trait loci (QTL) and $\mathbf{u}_i$ is an $m \times 1$ vector of the additive effects of the QTL associated with markers that affect the ith trait. It is assumed that $\boldsymbol{\gamma}_i$ has multivariate normal distribution with null mean and covariance matrix $\boldsymbol{\Phi}\sigma^2_{\gamma_i}$, where $\sigma^2_{\gamma_i}$ is the additive genomic variance of $\boldsymbol{\gamma}_i$ and $\boldsymbol{\Phi} = \mathbf{X}t(\mathbf{X})/\pi$ is the $g \times g$ additive genomic relationship matrix between individuals, where g denotes the number of genotypes and $t(\mathbf{X})$ is the transpose of matrix $\mathbf{X}$; $\pi = \sum_{q=1}^{m} 2p_q(1-p_q)$ in an F_2 population, and $\pi = \sum_{q=1}^{m} 4p_q(1-p_q)$ in a double haploid population.

In the genomics selection context, the covariance between $\boldsymbol{\gamma}_i$ and $\mathbf{g}_i$ (Supplementary Equation **1**) is equal[6] to $\sigma^2_{\gamma_i}$, therefore, the covariance matrix between $\boldsymbol{\gamma}$ and the transpose of $\mathbf{g}$, $t(\mathbf{g}) = [t(\mathbf{g}_1) \quad t(\mathbf{g}_2) \quad \cdots \quad t(\mathbf{g}_t)]$, is

$$\boldsymbol{\Gamma} = cov(\boldsymbol{\gamma}, t(\mathbf{g})) = var(\boldsymbol{\gamma}) = \{\sigma_{ij}\}, \quad \textbf{(9.2)}$$

a matrix of size $t \times t$ where σ_{ij} is the additive genomic covariance of $\boldsymbol{\gamma}_i$ and $\boldsymbol{\gamma}_j$ ($j, i = 1, 2, \ldots, t$). By this last result, after the first selection cycle (in which there are phenotypic and marker information), it is usually assumed that

$$\mathbf{G} = \boldsymbol{\Gamma}, \qquad \textbf{(9.3)}$$

because, in the genomics selection context, in cycle two, breeders only have markers information.

By Supplementary Equation **2** and by the earlier results, the quadratic genomic selection index can be written as

$$I_{qg} = t(\boldsymbol{\theta})\boldsymbol{\gamma} + t(\boldsymbol{\gamma})\mathbf{D}\boldsymbol{\gamma}, \qquad \textbf{(10)}$$

where $t(\boldsymbol{\theta}) = [\theta_1 \quad \theta_2 \quad \ldots \quad \theta_t]$ is the transpose of the vector of linear coefficients $\boldsymbol{\theta}$ and

$$\mathbf{D} = \begin{bmatrix} d_{11} & 0.5d_{12} & \cdots & 0.5d_{1t} \\ 0.5d_{12} & d_{22} & \cdots & 0.5d_{2t} \\ \vdots & \vdots & \ddots & \vdots \\ 0.5d_{1t} & 0.5d_{2t} & \ldots & d_{tt} \end{bmatrix}$$

is a matrix where the i^{th}diagonal values d_{ii} ($i = 1, 2, \ldots, t$) is the index weight for the square value of $\boldsymbol{\gamma}_i$ and $0.5d_{ij}$ ($i, j = 1, 2, \ldots, t$) is the index weight for the cross products between $\boldsymbol{\gamma}_i$ and $\boldsymbol{\gamma}_j$, in a similar manner as for matrix **B** (Supplementary Equation **2**). The expectation and variance of I_{qg}, and the covariance between $I_{qg}$and H_q are $E(I_{qg}) = tr(\mathbf{D}\boldsymbol{\Gamma})$, $Var(I_{qg}) = t(\boldsymbol{\theta})\boldsymbol{\Gamma}\boldsymbol{\theta} + 2tr[\mathbf{D}\boldsymbol{\Gamma}\mathbf{D}\boldsymbol{\Gamma}]$, and $Cov(H_q, I_{qg}) = t(\boldsymbol{\theta})\boldsymbol{\Gamma}\boldsymbol{\theta} + 2tr(\mathbf{D}\boldsymbol{\Gamma}\mathbf{W}\boldsymbol{\Gamma})$, respectively.

QGSI mean square prediction error (MSPE)

The QGSI MSPE can be written as

$$\Pi_g = E\{[I_{qg} - E(I_{qg})] - [H_q - E(H_q)]\}^2$$
$$= E\{[I_{qg} - E(I_{qg})]^2\} + E\{[H_q - E(H_q)]^2\} - 2E\{[I_{qg} - E(I_{qg})][H_q - E(H_q)]\}.$$

Using basic algebra, it is possible to show that

$E\{[I_{qg}-E(I_{qg})]^2\}=t(\boldsymbol{\theta})\boldsymbol{\Gamma\theta}+2tr(\mathbf{D\Gamma D\Gamma})$,

$E\{[H_q-E(H_q)]^2\}=t(\mathbf{w})\mathbf{Gw}+2tr(\mathbf{WGWG})$,

and

$-2E\{[I_{qg}-E(I_{qg})][H_q-E(H_q)]=-2t(\boldsymbol{\theta})\boldsymbol{\Gamma}\mathbf{w}-4tr(\mathbf{D\Gamma WG})$,

from where the QGSI MSPE is equal to

$$\Pi_G=t(\mathbf{w})\mathbf{Gw}+2tr(\mathbf{D\Gamma D\Gamma})+2tr(\mathbf{WGWG})+t(\boldsymbol{\theta})\boldsymbol{\Gamma\theta}-2t(\boldsymbol{\theta})\boldsymbol{\Gamma}\mathbf{w}-4tr(\mathbf{D\Gamma WG}) \quad \mathbf{(11)}$$

Minimizing the QGSI mean square prediction error

The firsts partial derivatives of Supplementary Equation **11** with respect to $\boldsymbol{\theta}$ and $\mathbf{D}$, are, respectively,

$\frac{\partial}{\partial\boldsymbol{\theta}}\Pi_G=\boldsymbol{\Gamma\theta}-\boldsymbol{\Gamma}\mathbf{w}=\mathbf{0}$ and $\frac{\partial}{\partial\mathbf{D}}\Pi_G=4\mathbf{\Gamma D\Gamma}-4\mathbf{GW\Gamma}=\mathbf{O}$,

therefore, by Supplementary Equation **9.2** and **9.3**,

$$\boldsymbol{\theta}=\mathbf{w} \text{ and } \mathbf{D}=\mathbf{W}. \qquad \mathbf{(12)}$$

In addition, note that the second partial derivatives of Supplementary Equation **11** with respect to $\boldsymbol{\theta}$ and $\mathbf{D}$ are $2\boldsymbol{\Gamma}$and $4\boldsymbol{\Gamma\Gamma}$, respectively. This means that, in effect, Supplementary Equation **12** results minimize Π_G.

By Supplementary Equation **12** and the assumption $\mathbf{G}=\boldsymbol{\Gamma}$ (Supplementary Equation **9.3**), the minimized Π_G value is

$$\Pi_G=0. \qquad \mathbf{(13)}$$

In theory, Supplementary Equation **13** is the desirable expected value of Π_G when this is minimized; however, in practice, $\mathbf{G}=\boldsymbol{\Gamma}$ only in the asymptotic context, when the number of markers tend to infinite. This means that in genomic selection context the true prediction error variance always will be higher than the estimated prediction error variance.

The correlation between $\mathrm{I_{qg}}$ and $\mathrm{H_q}$

According with Supplementary Equation **6**, a good measure of the relationship between $\mathrm{I_{qg}}$ and $\mathrm{H_q}$ when $\boldsymbol{\theta} = \mathbf{w}$ and $\mathbf{D} = \mathbf{W}$, is the square correlation between them, that is,

$$\rho^2_{H_q I_{qg}} = \frac{\mathrm{t}(\mathbf{w})\boldsymbol{\Gamma}\mathbf{w} + 2\mathrm{tr}(\mathbf{W}\boldsymbol{\Gamma}\mathbf{W}\boldsymbol{\Gamma})}{\mathrm{t}(\mathbf{w})\mathbf{G}\mathbf{w} + 2\mathrm{tr}[\mathbf{W}\mathbf{G}\mathbf{W}\mathbf{G}]}, \qquad \textbf{(14)}$$

where all the parameters were defined earlier. Note that Supplementary Equation **14** only can be estimated with simulated data, and when $\boldsymbol{\Gamma} = \mathbf{G}$, this equation is equal to 1.0.

The QGSI selection response and expected genetic gain per trait

When $\boldsymbol{\theta} = \mathbf{w}$ and $\mathbf{D} = \mathbf{W}$, the QGSI selection response and expected genetic gain per trait are

$$R_{qg} = k\sqrt{\mathrm{t}(\mathbf{w})\boldsymbol{\Gamma}\mathbf{w} + 2\mathrm{tr}[\mathbf{W}\boldsymbol{\Gamma}\mathbf{W}\boldsymbol{\Gamma}]}\,, \qquad \textbf{(15)}$$

and

$$\mathbf{E}_{qg} = \mathrm{k}\frac{\boldsymbol{\Gamma}\mathbf{w}}{\sqrt{\mathrm{t}(\mathbf{w})\boldsymbol{\Gamma}\mathbf{w}}}, \qquad \textbf{(16)}$$

respectively, where k is the selection intensity of the QGSI whereas the other terms were defined earlier. Supplementary Equation **14-16** indicate that the LGSI and the QGSI are related, and the only difference between them is the trace of the quadratic terms.

Supplementary Method 3. Maximum likelihood and Bayesian parameter estimation

Estimating matrices P and G

By the maximum likelihood (ML) parameter estimation invariance property, the estimators of the QPSI, QGSI, LPSI, and LGSI parameters, are all ML estimators[7]. Maximum likelihood methods to estimate the genotypic and phenotypic variance and covariance have been described[8]. We used the linear mixed model effects[8] and the R-package lme4[9] to estimate the genotypic **G** (**C**) and residual ($\boldsymbol{\Xi}$) covariance matrices, from where the estimator (**S**) of the phenotypic covariance matrix (**P**) was

$$\mathbf{S} = \mathbf{C} + \frac{1}{n_e n_r}\widehat{\boldsymbol{\Xi}}\,, \qquad \mathbf{(17)}$$

where n_e denotes the number of environments where the population of genotypic values were evaluated, and n_r denotes the number of repetitions of each genotype within each population.

Estimating the QPSI and LPSI parameters

We estimated the QPSI and LPSI inserting **S** and **C** into Supplementary Equation **4-8**. For example, the two parameters in Supplementary Equation **4** were estimated as

$$\hat{\mathbf{b}} = \mathbf{S}^{-1}\mathbf{C}\mathbf{w} \text{ and } \widehat{\mathbf{B}} = \mathbf{S}^{-1}\mathbf{C}\mathbf{W}\mathbf{C}\mathbf{S}^{-1}, \qquad \mathbf{(18)}$$

where $\mathbf{S}^{-1}$ is a ML estimator of the inverse of matrix **S.**

Estimating matrix *Γ*

Methods to estimate matrix **Γ** (Supplementary Equation **9.2**) has been described[5]. In this method the covariances that conform matrix **Γ** are estimated as

$$\widehat{\mathrm{cov}}(\boldsymbol{\gamma}_i, \boldsymbol{\gamma}_j) = \frac{1}{g}\mathrm{t}(\hat{\boldsymbol{\gamma}}_i)\boldsymbol{\Phi}^{-1}\hat{\boldsymbol{\gamma}}_j, \text{ for } i \neq j, \qquad \mathbf{(19.1)}$$

or

$$\widehat{\text{cov}}(\boldsymbol{\gamma}_{\text{i}}, \boldsymbol{\gamma}_{\text{j}}) = \frac{1}{\text{g}} \text{t}(\hat{\boldsymbol{\gamma}}_{\text{i}})\hat{\boldsymbol{\gamma}}_{\text{j}}, \text{ for i} \neq \text{j}, \qquad (\mathbf{19.2})$$

where $\text{t}(\hat{\boldsymbol{\gamma}}_{\text{i}})$ denotes the transpose of the ith vector of genomic estimated breeding values (GEBV) $\hat{\boldsymbol{\gamma}}_i$, g is the number of genotypes in the dataset, and $\boldsymbol{\Phi}^{-1}$ is the inverse of the g × g additive genomic relationship matrix $\boldsymbol{\Phi} = \mathbf{X}\text{t}(\mathbf{X})/\pi$ between individuals defined earlier. In the present work, we estimated matrix $\boldsymbol{\Gamma}$ using Supplementary Equation **19.2**.

Estimating trait and GEBV, and index heritability

For each trait, the narrow-sense heritability (also called realized heritability) was estimated as the diagonal matrix of the elements of matrix $\mathbf{C}$ ($\mathbf{D}_{\mathbf{C}}$) multiplied by the inverse matrix of the diagonal elements of matrix $\mathbf{S}$ ($\mathbf{D}_{\mathbf{S}}^{-1}$)

$$\hat{\mathbf{h}}^2_{\text{Traits}} = \mathbf{D}_{\mathbf{S}}^{-1}\mathbf{D}_{\mathbf{C}}\,, \qquad (\mathbf{20.1})$$

where $\hat{\mathbf{h}}^2_{\text{Traits}}$ is a vector of traits heritability. In a similar manner, for each genomic estimated breeding values (GEBV), we estimated its heritability as

$$\hat{\mathbf{h}}^2_{\text{GEBV}} = \mathbf{D}_{\mathbf{S}}^{-1}\mathbf{D}_{\hat{\boldsymbol{\Gamma}}}, \quad (\mathbf{20.2})$$

where $\hat{\mathbf{h}}^2_{\text{GEBV}}$ is a vector of GEBV heritability, $\mathbf{D}_{\hat{\boldsymbol{\Gamma}}}$ is the diagonal matrix of the elements of matrix $\hat{\boldsymbol{\Gamma}}$ (Supplementary Equation **19.2**) whereas $\mathbf{D}_{\mathbf{S}}^{-1}$ is the inverse matrix of the diagonal elements of matrix **S**, which, in this case **S** was associated to the ten simulated datasets for genomic selection.

The index LPSI and LGSI[4] broad-sense heritability was estimated as

$$\widehat{\text{H}}^2_{\text{LPSI}} = \frac{\text{t}(\hat{\mathbf{b}})\mathbf{C}\hat{\mathbf{b}}}{\text{t}(\hat{\mathbf{b}})\mathbf{S}\hat{\mathbf{b}}}\,, \qquad (\mathbf{20.3})$$

and

$\widehat{H}^2_{LGSI} = \frac{t(\mathbf{w})\hat{\boldsymbol{\Gamma}}w}{t(\mathbf{w})\mathbf{S}w}$, **(20.4)**

respectively, where $t(\hat{\mathbf{b}})$ and $t(\mathbf{w})$ denote the transpose of vector $\hat{\mathbf{b}}$ and $\mathbf{w}$, and, in Supplementary Equation **20.2**, matrix **S** was associated to the ten simulated maize datasets for genomic selection. The heritability for the QPSI and QGSI are not defined until now.

Bayesian estimation

Multivariate mixed model without covariates[10] (GBLUP, RKHS with one kernel)

$$\mathbf{Y} = \mathbf{1}\mu' + \mathbf{U} + \Xi \quad \textbf{(21)}$$

where $\mathbf{U} \sim MN\ (\mathbf{0}, \boldsymbol{\Phi}, \mathbf{V})$ is an $n \times t$ matrix of random effects, and $\Xi \sim MN\ (\mathbf{0},\ V(\Xi))$ is an $n \times n$ matrix of residual random effects. Matrix $\boldsymbol{\Phi}$ is the $g \times g$ scale matrix modeling variance–covariance across rows of **U**, for example: A case could be that $\boldsymbol{\Phi} = \mathbf{A}$, with **A** the additive relationship matrix derived from pedigree relationship, or another case is when $\boldsymbol{\Phi} = \mathbf{X}t(\mathbf{X})/\pi$, with $\boldsymbol{\Phi}$ the centered and standardized marker matrix and π the number of markers. In general, $\boldsymbol{\Phi}$ denotes a kernel. **V** is the $t \times t$ scale matrix modeling variance–covariance across columns of **U** .

Gaussian kernel

The kernel $\boldsymbol{\Phi}$ can be built from centered and standardized markers. For the Gaussian kernel, a bandwidth h is selected:

$$\boldsymbol{\Phi}_h(\mathbf{m}_i, \mathbf{m}_j) = \exp\left\{-\frac{1}{h} \times \frac{\sum_{q=1}^{\pi}(m_{iq} - m_{jq})^2}{\pi}\right\}$$

Three bandwidths were used $h_1 = (1/M)1/5$, $h_2 = (1/M)$, $h_3 = (1/M)1/5$ where M is the median of the squared Euclidean distances between individuals using off-diagonal entries[10].

Sequence of fitted models

$$\mathbf{Y} = \mathbf{1}\mu' + \mathbf{U}_1 + \boldsymbol{\Xi} \text{ with } \mathbf{U}_1 \sim \text{MN}(\mathbf{0}, \boldsymbol{\Phi}_1, \mathbf{V}_1)\text{, using } \text{h}_1$$

$$\mathbf{Y} = \mathbf{1}\mu' + \mathbf{U}_2 + \boldsymbol{\Xi} \text{ with } \mathbf{U}_2 \sim \text{MN}(\mathbf{0}, \boldsymbol{\Phi}_2, \mathbf{V}_2)\text{, using } \text{h}_2$$

$$\mathbf{Y} = \mathbf{1}\mu' + \mathbf{U}_3 + \boldsymbol{\Xi} \text{ with } \mathbf{U}_3 \sim \text{MN}(\mathbf{0}, \boldsymbol{\Phi}_3, \mathbf{V}_3)\text{, using } \text{h}_3$$

Matrices $\mathbf{V}_1$, $\mathbf{V}_2$, $\mathbf{V}_3$ were estimated, yielding $\widehat{\mathbf{V}}_1$, $\widehat{\mathbf{V}}_2$, $\widehat{\mathbf{V}}_3$ and traces tr_1, tr_2, tr_3. A weighted Gaussian kernel was then constructed as

$$\overline{\boldsymbol{\Phi}} = \frac{tr_1}{tr_1 + tr_2 + tr_3}\boldsymbol{\Phi}_1 + \frac{tr_2}{tr_1 + tr_2 + tr_3}\boldsymbol{\Phi}_2 + \frac{tr_3}{tr_1 + tr_2 + tr_3}\boldsymbol{\Phi}_3$$

Thus, the final model is

$$\mathbf{Y} = \mathbf{1}\boldsymbol{\mu}' + \overline{\mathbf{U}} + \boldsymbol{\Xi},$$

with $\overline{\mathbf{U}} \sim \text{NM}(\mathbf{0}, \overline{\boldsymbol{\Phi}}, \overline{\mathbf{V}})$, from where $\widehat{\overline{\mathbf{V}}}$ (matrix of genetic variances and covariances) and the breeding values $\widehat{\overline{\mathbf{U}}}$, are obtained.

Model fitting with BGLR

The analyses were performed using the BGLR package[10] (multi-trait function). For fitting the two models above, 100,000 iterations were run; the first 50,000 were used as burn-in, and a thinning interval of 10 was applied.

Supplementary Method 4. Illustrative examples

Estimated LPSI parameters

The restricted maximum likelihood (REML) is one of the best methods to estimate **P** and **G**. Details to estimate **P** and **G** are given in Supplementarial Equation **17**. This equation was used in this work to estimates matatrices **P** (**S**) and **G** (**C**) in the simulated and real datasets. For real JMpop1 DTMA Mexico in cycle C_0, the REML estimates of **P**, **G**, and $\mathbf{P}^{-1}(\mathbf{S}^{-1})$ were

$$\mathbf{S} = \begin{bmatrix} 1.43 & 4.30 & 3.48 & 0.14 \\ 4.30 & 120.14 & 64.81 & 1.63 \\ 3.48 & 64.81 & 88.58 & -0.72 \\ 0.14 & 1.63 & -0.72 & 1.37 \end{bmatrix}, \quad \mathbf{S}^{-1} = \begin{bmatrix} 0.806 & -0.017 & -0.019 & -0.072 \\ -0.017 & 0.015 & -0.010 & -0.021 \\ -0.019 & -0.010 & 0.020 & 0.025 \\ -0.072 & -0.021 & 0.025 & 0.775 \end{bmatrix},$$

and $\mathbf{C} = \begin{bmatrix} 1.00 & 3.51 & 3.13 & 0.30 \\ 3.51 & 66.50 & 39.04 & 2.45 \\ 3.13 & 39.04 & 33.84 & 0.13 \\ 0.30 & 2.45 & 0.13 & 0.84 \end{bmatrix}$, respectively.

The estimated LPSI vector of coefficients was

$$\mathrm{t}(\hat{\mathbf{b}}) = \mathrm{t}(\mathbf{w})\mathbf{C}\mathbf{S}^{-1} = [2.602 \quad -0.224 \quad -0.011 \quad -0.088],$$

where $\mathrm{t}(\hat{\mathbf{b}})$and $\mathrm{t}(\mathbf{w})$ are are the tranpose of vector $\hat{\mathbf{b}}$ (Supplementary Equation **18**) and **w**, respectively; whereas the estimated LPSI was

$$\hat{\mathrm{I}}_{\mathrm{i}} = 2.698(\mathrm{GY} - \overline{\mathrm{GY}}) - 0.227(\mathrm{PHT} - \overline{\mathrm{PHT}}) - 0.040(\mathrm{EHT} - \overline{\mathrm{EHT}}) - 0.089(\mathrm{AD} - \overline{\mathrm{AD}})$$

where $\overline{\mathrm{GY}}$, $\overline{\mathrm{PHT}}$, $\overline{\mathrm{EHT}}$, and $\overline{\mathrm{AD}}$ are the averages of traits GY, PHT, EHT, and AD, respectively. For a selection intensity of 10% (k = 1.755), the estimated selection response and the square of the estimated correlation were

$\hat{\mathrm{R}} = \mathrm{k}\sqrt{\mathrm{t}(\hat{\mathbf{b}})\mathbf{S}\hat{\mathbf{b}}} = 1.755\mathrm{k}\sqrt{11.372} = 5.918$ and $\hat{\rho}^2_{\mathrm{H}\hat{\mathrm{I}}} = \frac{11.372}{20.620} = 0.552$, respectively, where $\mathrm{t}(\hat{\mathbf{b}})$ was deffined earlier; whereas the vector of expected genetic gain per trait and the estimated LPSI mean square prediction error (MSPE, $\hat{\sigma}^2_{\mathrm{e}}$) were

$$\hat{\mathbf{E}} = (1.755)\frac{\mathrm{t}(\hat{\mathbf{b}})\mathbf{C}}{\sqrt{\mathrm{t}(\hat{\mathbf{b}})\mathbf{S}\hat{\mathbf{b}}}} = [1.418 \quad -8.622 \quad -3.303 \quad -0.064]$$

and

$\widehat{\sigma}_e^2 = \widehat{\sigma}_H^2 - \widehat{\sigma}_I^2 = 20.620 - 11.372 = 9.247$,

respectively, where $\widehat{\sigma}_H^2$ is the estimated variance of the linear net genetic merit and $\widehat{\sigma}_I^2$ is the estimated LPSI variance.

Estimated QPSI parameters

For the real dataset 3 in cycle C_0, the estimated **B** ($\widehat{\mathbf{B}}$) matrix (Supplementary Equation **4** and **18**) was,

$$\widehat{\mathbf{B}}_0 = \begin{bmatrix} -0.518 & -0.5105 & -0.2769 & -0.2769 \\ -0.5105 & -0.0751 & -0.0405 & -0.1126 \\ -0.2769 & -0.0405 & -0.0337 & -0.1126 \\ -0.2769 & -0.1126 & -0.1126 & -1.1124 \end{bmatrix}.$$

Matrix **W** (Supplementary Equation **1**) was obtained from the vector of economic weights $\mathbf{w}' = [5 \quad -0.3 \quad -0.3 \quad -1]$ as

$$\mathbf{W} = \begin{bmatrix} 5.000 & -0.750 & -0.750 & -2.500 \\ -0.750 & -0.300 & 0.045 & 0.150 \\ -0.750 & 0.045 & -0.300 & 0.150 \\ -2.500 & 0.150 & 0.150 & -1.000 \end{bmatrix},$$ from where the estimated QPSI (Supplementary Equation **2**) was

$$\hat{\mathrm{I}}_{qi} = \hat{\mathrm{I}}_i + \mathrm{t}(\mathbf{y})\widehat{\mathbf{B}}\mathbf{y},$$

where $\hat{\mathrm{I}}_i$ (centered respect its average) is the estimated LPSI defined earlier, and $\mathrm{t}(\mathbf{y})$ is the transpose of vector **y** which containts the traits values: GY, PHT, EHT, and AD centered respect to $\overline{\mathrm{GY}}$, $\overline{\mathrm{PHT}}$, $\overline{\mathrm{EHT}}$, and $\overline{\mathrm{AD}}$, which were defined earlier. For $k = 1.755$, the estimated QPSI square correlation and selection response (Supplementary Equation **6** and **7**) were

$$\hat{\rho}^2_{\mathrm{H_q}\hat{\mathrm{I}}_q} = \frac{\mathrm{t}(\hat{\mathbf{b}})\mathbf{S}\hat{\mathbf{b}}+2\mathrm{tr}[\widehat{\mathbf{B}}\mathbf{SWC}]}{\mathrm{t}(\hat{\mathbf{b}})\mathbf{C}\mathbf{w}+2\mathrm{tr}[\mathbf{WCWC}]} = 0.50 \text{ and } \widehat{\mathrm{R}}_q = \mathrm{k}\sqrt{\mathrm{t}(\hat{\mathbf{b}})\mathbf{S}\hat{\mathbf{b}} + 2\mathrm{tr}[\widehat{\mathbf{B}}\mathbf{SWC}]} = 59.714,$$

respectively, where $tr[.]$ denote the trace of the product of the estimated matrices, whereas $\mathrm{t}(\hat{\mathbf{b}})\mathbf{S}\hat{\mathbf{b}}$ is the estimated variance of the LPSI, and $\mathrm{t}(\mathbf{w})\mathbf{C}\mathbf{w}$ is the estimated variance of the linear net genetic merit. Note that the estimated QPSI vector of expected genetic gain per trait is the same that the LPSI vector of expected genetic gain per trait described earlier (Supplementary Equation

8). Additional results for JMpop1 DTMA Mexico dataset and JMpop1 DTMA Zimbabwe dataset are in Table 3 and 4.

Estimated LGSI parameters

In Supplementary Equation **19.1** and **19.2**, we described two methods to estimate matrix $\mathbf{\Gamma}$. For JMpop1 DTMA Mexico dataset in cycle 1 (C_1), the estimated $\mathbf{\Gamma}$ ($\hat{\mathbf{\Gamma}}$) matrix is

$$\hat{\mathbf{\Gamma}} = \begin{bmatrix} 0.523 & 2.500 & 1.810 & 0.2610 \\ 2.500 & 24.226 & 15.125 & 1.512 \\ 1.810 & 15.125 & 11.171 & 0.590 \\ 0.2610 & 1.512 & 0.590 & 0.349 \end{bmatrix}.$$

The estimated LGSI (centered respect its average) was

$$\hat{I}_{gi} = 5 \times GEBV_{GY} - 0.3 \times GEBV_{PHT} - 0.3 \times GEBV_{EHT} - 1 \times GEBV_{AD}$$

where all the genomic estimated breeding values ($GEBV_{GY}$, $GEBV_{PHT}$, $GEBV_{EHT}$, and $GEBV_{AD}$) of traits GY, PHT, EHT, and AD were centered respect their average ($\overline{GEBV_{GY}}$, $\overline{GEBV_{PHT}}$, $\overline{GEBV_{EHT}}$, and $\overline{GEBV_{AD}}$). For $k = 1.755$, the estimated LGSI selection response and the vector of expected genetic gain per trait were

$$\hat{R}_g = k\sqrt{t(\mathbf{w})\hat{\mathbf{\Gamma}}\mathbf{w}} = 3.948$$

and

$$\hat{\mathbf{E}}_g' = k\frac{t(\mathbf{w})\hat{\mathbf{\Gamma}}}{\sqrt{t(\mathbf{w})\hat{\mathbf{\Gamma}}\mathbf{w}}} = [0.828 \quad -0.654 \quad 0.446 \quad 0.254],$$

respectively.

Estimated QGSI parameters

For the real JMpop1 DTMA Mexico dataset in cycle C_1 and Suplementary Equation **2**, the estimated QGSI (centered respect its average) was

$$\hat{I}_{qgi} = \hat{I}_{gi} + t(\hat{\boldsymbol{\gamma}})\mathbf{W}\hat{\boldsymbol{\gamma}},$$

where $\hat{I}_{gi}$ is the estimated LGSI defined earlier, and $t(\hat{\boldsymbol{\gamma}})$ is transpose of vector $\hat{\boldsymbol{\gamma}}$ which containts the genomic estimated breeding values ($GEBV_{GY}$, $GEBV_{PHT}$, $GEBV_{EHT}$, and $GEBV_{AD}$) of the traits GY, PHT, EHT, centered respect their average as deffined earlier. In additon, for $k = 1.755$, the estimated QGSI selection response (Supplementary Equation **15**) was

$$\widehat{R}_{qg} = k\sqrt{t(\mathbf{w})\hat{\boldsymbol{\Gamma}}\mathbf{w} + 2tr[\mathbf{W}\hat{\boldsymbol{\Gamma}}\mathbf{W}\hat{\boldsymbol{\Gamma}}]} = 35.815$$

where $tr[.]$ denote the trace of the product of the estimated matrices, whereas $t(\mathbf{w})\hat{\boldsymbol{\Gamma}}\mathbf{w}$ is the estimated variance of the LGSI. The estimated QGSI vector of expected genetic gain per trait is the same that the LGSI vector of expected genetic gain per trait described earlier. The results associated to C_2 for JMpop1 DTMA Mexico dataset, and those associated JMpop1 DTMA Zimbabwe dataset are in Table 3 and 4.

Supplementary Table 1. One-side ten statistics Shapiro-Wilk normality test and test *p*-values.

	Quadratic phenotypic selection index					
	Shapiro-Wilk test			*p*-values		
Cycle	Distribution of H_q and QPSI	Residuals	QPSI	Distribution of H_q and QPSI	Residuals	QPSI
1	0.994	0.996	0.996	0.053	0.196	0.268
2	0.996	0.996	0.995	0.198	0.207	0.106
3	0.996	0.998	0.997	0.184	0.855	0.651
4	0.998	0.996	0.998	0.682	0.242	0.818
5	0.997	0.998	0.997	0.542	0.778	0.454
6	0.996	0.997	0.996	0.251	0.626	0.247
7	0.997	0.993	0.998	0.623	0.642	0.766
8	0.998	0.999	0.998	0.868	0.959	0.766
9	0.998	0.999	0.998	0.929	0.959	0.766
10	0.997	0.999	0.998	0.519	0.959	0.766
Average	0.997	0.997	0.997	0.485	0.642	0.561
	Quadratic genomic selection index					
	Shapiro-Wilk test			*p*-values		
Cycle	Distribution of H_q and QGSI	Residuals	QGSI	Distribution of H_q and QGSI	Residuals	QGSI
1	0.997	0.999	0.994	0.504	0.980	0.041
2	0.998	0.994	0.998	0.912	0.061	0.953
3	0.996	0.997	0.996	0.383	0.626	0.369
4	0.998	0.996	0.996	0.848	0.359	0.304
5	0.998	0.998	0.998	0.912	0.712	0.984
6	0.994	0.994	0.997	0.035	0.033	0.421
7	0.997	0.996	0.997	0.584	0.204	0.558
8	0.997	0.996	0.997	0.586	0.204	0.558
9	0.996	0.996	0.997	0.286	0.204	0.558
10	0.996	0.996	0.997	0.187	0.204	0.558
Average	0.997	0.996	0.997	0.524	0.359	0.531

Results were obtained for ten simulated selection cycles testing the bivariate normality of the joint distribution of the true quadratic net genetic merit (H_q) and the estimated quadratic phenotypic and genomic selection index (QPSI, QGSI, respectively) and the univariate normality of the residuals and QPSI. The criteria to accept or reject the normality assumption (null hypothesis) was the *p*-value. A low *p*-value means that the null hypothesis of normality is false.

Supplementary Table 2. One-side (Shapiro-Wilk (SW), Henze-Wagner, Henze-Zirkler, and Royston) and two-side (Mardia) five test statistics and their *p*-values.

Joint multivariate normality tests for four simulated maize traits in ten cycles

	Tests Statistics						*p*-values					
		Mardia		Henze	Henze			Mardia		Henze	Henze	
Cycle	SW	Skewness	Kurtosis	Wagner	Zirkler	Royston	SW	Skewness	Kurtosis	Wagner	Zirkler	Royston
1	0.994	35.9	0.96	0.86	1.02	20.32	0.04	0.02	0.34	0.01	0.07	0.00
2	0.993	10.5	-0.45	0.63	0.90	1.75	0.02	0.96	0.65	0.33	0.45	0.78
3	0.991	20.2	-0.37	0.43	0.79	4.09	0.00	0.45	0.71	0.96	0.90	0.39
4	0.996	29.0	-0.17	0.67	0.91	0.47	0.32	0.09	0.86	0.22	0.41	0.98
5	0.992	19.7	0.69	0.60	0.85	2.72	0.01	0.48	0.49	0.43	0.70	0.60
6	0.996	12.1	-1.01	0.42	0.77	2.30	0.24	0.91	0.31	0.97	0.94	0.68
7	0.996	22.1	0.93	0.82	1.03	7.27	0.24	0.34	0.35	0.03	0.07	0.12
8	0.996	19.2	-0.67	0.80	1.02	3.70	0.24	0.51	0.51	0.04	0.08	0.45
9	0.995	16.4	0.17	0.48	0.79	1.14	0.11	0.69	0.87	0.86	0.89	0.89
10	0.996	24.9	-0.79	0.57	0.96	1.30	0.24	0.20	0.43	0.57	0.22	0.86
Average	0.995	21.0	-0.07	0.63	0.90	4.51	0.15	0.46	0.55	0.44	0.47	0.58

Joint multivariate normality tests for four simulated maize GEBV in ten cycle

	Tests Statistics						*p*-values					
		Mardia		Henze	Henze			Mardia		Henze	Henze	
Cycle	SW	Skewness	Kurtosis	Wagner	Zirkler	Royston	SW	Skewness	Kurtosis	Wagner	Zirkler	Royston
1	0.984	49.9	0.69	1.03	1.06	23.58	0.00	0.00	0.49	0.00	0.04	0.00
2	0.998	13.5	-1.92	0.60	0.88	1.87	0.68	0.86	0.06	0.44	0.54	0.74
3	0.996	28.9	-1.11	0.64	0.96	5.39	0.33	0.09	0.27	0.31	0.20	0.24
4	0.992	34.2	-0.03	0.59	0.84	4.40	0.01	0.02	0.97	0.46	0.73	0.29
5	0.995	24.9	-0.07	0.65	0.96	2.34	0.15	0.21	0.94	0.28	0.21	0.65
6	0.993	22.9	-0.31	0.69	1.00	3.91	0.02	0.29	0.75	0.16	0.11	0.42
7	0.996	31.3	-1.61	0.90	1.04	6.73	0.20	0.05	0.11	0.01	0.06	0.14
8	0.996	26.4	-1.19	0.56	0.85	1.42	0.16	0.15	0.23	0.58	0.69	0.84
9	0.995	26.3	-0.70	0.50	0.86	6.78	0.10	0.16	0.49	0.82	0.64	0.15
10	0.996	22.3	-0.30	0.42	0.72	3.29	0.25	0.33	0.77	0.97	0.99	0.51
Average	0.994	28.0	-0.66	0.66	0.92	5.97	0.19	0.22	0.51	0.40	0.42	0.40

Results are the tests of joint multivariate normality of four traits and four genomics estimated breeding values (GEBV) in ten simulated maize cycles datasets. The criteria to accept or reject the normality assumption (Null hypothesis) was the *p*-value. A low p-value means that the null hypothesis of normality is false.

Supplementary Table 3. One-side (Shapiro-Wilk (SW), Henze-Wagner, Henze-Zirkler, and Royston) and two- side (Mardia) five test statistics and *p*-values.

Joint multivariate normality tests for four real maize traits

		Tests statistics						*p*-values					
	Training		Mardia		Henze	Henze			Mardia		Henze	Henze	
set	Population	SW	Skewness	Kurtosis	Wagner	Zirkler	Royston	SW	Skewness	Kurtosis	Wagner	Zirkler	Royston
JDMexico	0	0.89	246.26	15.17	2.05	1.62	95.09	0	0	0	0	0	0
JDZimbabwe	0	0.97	59.08	2.82	0.66	0.85	31.32	0	0	0	0.23	0.52	0
Average		0.93	152.67	9.00	1.35	1.24	63.21	0	0	0	0.12	0.26	0

Joint multivariate normality tests for four real maize GEBVs

		Test statistics						*p*-values					
	Training		Mardia		Henze	Henze			Mardia		Henze	Henze	
Set	Populations	SW	Skewness	Kurtosis	Wagner	Zirkler	Royston	SW	Skewness	Kurtosis	Wagner	Zirkler	Royston
JDMexico	1	0.99	35.21	1.40	0.92	0.99	22.41	0	0.02	0.16	0.00	0.11	0
	2	0.99	25.75	-0.80	0.63	0.89	1.55	0.22	0.17	0.42	0.33	0.41	0.71
JDZimbabwe	1	0.99	14.89	-0.56	0.59	1.02	3.60	0.01	0.78	0.57	0.47	0.06	0.40
	2	0.99	19.02	-0.33	0.64	0.89	4.36	0.14	0.52	0.74	0.31	0.38	0.32
Average		0.99	23.72	-0.07	0.70	0.95	7.98	0.09	0.37	0.47	0.28	0.24	0.36

Joint multivariate normality test for three and two real wheat traits

	Tests statistics						*p*-values					
		Mardia		Henze	Henze			Mardia		Henze	Henze	
Environments	SW	Skewness	Kurtosis	Wagner	Zirkler	Royston	SW	Skewness	Kurtosis	Wagner	Zirkler	Royston
CENEB-BED2IR	0.94	16.66	2.22	0.46	0.61	16.21	0.01	0.08	0.03	0.43	0.61	0
CIANO-BED-5IR	0.96	10.19	-0.62	0.38	0.58	3.63	0.12	0.42	0.53	0.69	0.69	0.30
CENEB-FLATDRIP	0.94	16.66	2.22	0.46	0.61	16.21	0.01	0.08	0.03	0.43	0.61	0
All environments	0.98	4.42	-0.23	0.36	0.58	5.31	0.48	0.93	0.82	0.76	0.70	0.15
SAWYT-27	0.90	20.27	1.59	0.45	0.58	10.97	0.00	0.03	0.11	0.47	0.66	0.01
SAWYT-28	0.95	9.01	1.02	0.33	0.53	5.73	0.03	0.06	0.31	0.32	0.37	0.06
Average	0.94	12.87	1.03	0.41	0.58	9.68	0.11	0.27	0.30	0.52	0.61	0.09

Joint multivariate normality test for three and two real wheat GEBVs

	Test statistics						*p*-values					
		Mardia		Henze	Henze			Mardia		Henze	Henze	
Environments	SW	Skewness	Kurtosis	Wagner	Zirkler	Royston	SW	Skewness	Kurtosis	Wagner	Zirkler	Royston
CENEB-BED2IR	0.94	14.96	0.39	0.46	0.65	10.95	0.02	0.13	0.69	0.44	0.48	0.01
CIANO-BED-5IR	0.98	4.42	-0.93	0.47	0.77	2.48	0.43	0.93	0.35	0.38	0.21	0.47
CENEB-FLATDRIP	0.98	27.09	-0.60	1.45	1.75	7.06	0.43	0	0.55	0	0	0.03
All environments	0.98	21.75	-1.15	1.00	1.26	6.09	0.49	0.02	0.25	0	0	0.10
SAWYT-27	0.89	20.95	2.07	0.47	0.61	6.86	0.00	0.02	0.04	0.41	0.57	0.08
SAWYT-28	0.97	5.36	-0.15	0.22	0.40	2.73	0.24	0.25	0.88	0.68	0.66	0.25
Average	0.95	15.76	-0.06	0.68	0.91	6.03	0.27	0.23	0.46	0.32	0.32	0.16

In this table we used four traits and GEBV for the normality test whereas for wheat we used three traits and its GEBV in the tests. The criteria to accept or reject the normality assumption (Null hypothesis) was the *p*-value. A low *p*-value means that the null hypothesis of normality is false.

Supplementary Table 4. Broad-sense heritability for real maize and wheat traits, and linear phenotypic selection index (LPSI).

Set	Training Population	Real maize trait heritability				
		GY	EHT	PHT	AD	LPSI
JDMexico	0	0.728	0.616	0.455	0.682	0.570
JDZimbabwe	0	0.160	0.500	0.461	0.512	0.713
Average		0.444	0.558	0.458	0.597	0.641

Environments	Real wheat trait heritability				LPSI
	GY	HD	PHT	AD	
CENEB-BED2IR	0.292	0.867	0.658	*	0.547
CIANO-BED-5IR	0.292	0.895	0.639	*	0.449
CENEB-FLATDRIP	0.024	0.927	0.337	*	0.907
All environments	0.082	0.887	0.458	*	0.654
SAWYT-27	0.727	0.957	0.745	*	0.634
SAWYT-28	0.362	0.947	*	*	0.760

*Non results for that trait

The results correspond to four real maize traits (GY, EHT, PHT, AD) and three real wheat traits (GY, HD, PHT).

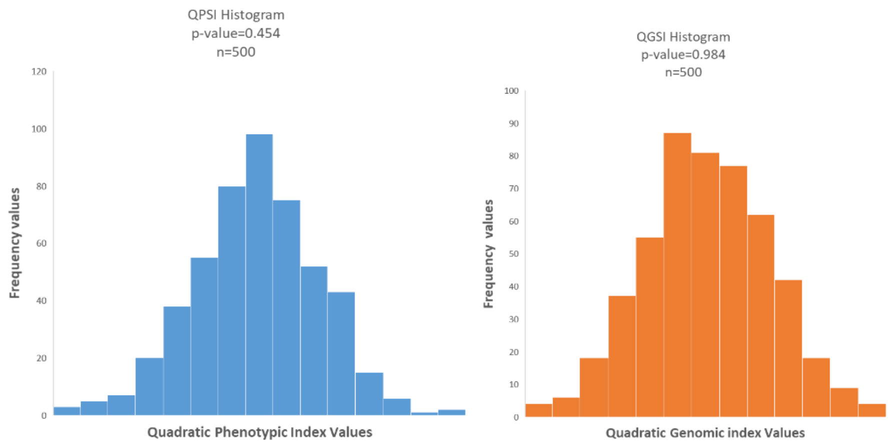

Supplementary Figure 1. **Distribution diagnostics and normality tests for quadratic and genomic selection index (QPSI and QGSI, respectively).**

Supplementary references

1. Wilton, J. W., Evans, A. & Van Vleck, L. D. Selection indices for quadratic models of total merit. *Biometrics* **24**, 937–949 (1968).
2. Groen, A. F., Meuwissen, T. H. E., Vollema, A. R. & Brascamp, E. W. A comparison of alternative index procedures for multiple-generation selection on non-linear profit. *Animal Production* **59**, 1–9 (1994).
3. Smith, H. F. A discriminant function for plant selection. *Annals of Eugenics* 7, 240–250 (1936).
4. Cerón-Rojas, J. J. & Crossa, J. *Linear Selection Indices in Modern Plant Breeding* Chs. 2 and 5 (Springer, The Netherlands, 2018).
5. Cerón-Rojas, J. J., Crossa, J., Arief, V. N., Basford, K., Rutkoski, J., Jarquín, D., Alvarado, G., Beyene, Y., Semagn, K., & DeLacy, I. A genomic selection index applied to simulated and real data. *Genes/Genomes/Genetics* **5**, 2155–2164 (2015).
6. Dekkers, J. C. M. Prediction of response to marker-assisted and genomic selection using selection index theory. *Journal of Animal Breeding and Genetics* **124**, 331–341 (2007).
7. Giri, N.C. *Multivariate Statistical Analysis* Ch.5 (Marcel Dekker, New York, 2004).
8. Lynch, M. & Walsh, B. *Genetics and Analysis of Quantitative Traits* Ch.27 (Sinauer, Sunderland, 1998).
9. Bates, D., Mächler , M., Bolker , B. M. & Walker, S. C. Fitting Linear Mixed-Effects Models Using lme4. *Journal of Statistical Software* **67**, 1-48 (2015).
10. Pérez, P. & de los Campos, G. Multitrait Bayesian shrinkage and variable selection models with the BGLR-R package. *Genetics* **222**, iyac112 (2022).
